# Subacute COVID-19 Infection Presenting as Indolent Large Pericardial Effusion

**DOI:** 10.7759/cureus.10769

**Published:** 2020-10-02

**Authors:** Chidinma Ejikeme, Melissa Gonzalez, Sherif Elkattawy, Ramez Alyacoub, Clark Sherer

**Affiliations:** 1 Internal Medicine, Rutgers New Jersey Medical School/Trinitas Regional Medical Center, Elizabeth, USA; 2 Infectious Disease, Trinitas Regional Medical Center, Elizabeth, USA

**Keywords:** covid 19, pericardial, effusion

## Abstract

Reports of complications as a result of COVID-19 infection are emerging since the virus became a pandemic. Although not fully understood, reports show that the COVID-19 virus has shown acute pericardial involvement resulting from this infection. It can cause a wide range of manifestations from minimal effusion to large effusion with tamponade; however, there is little or no data on an indolent course of COVID-19 infection and its resulting manifestations. Here we describe a patient who had minimal disease symptoms for weeks, resulting in sizeable pericardial effusion formation.

## Introduction

COVID-19 is a global pandemic first described in Wuhan, China. Research studies from China described respiratory symptoms as the most common disease presentation [1]. Now widespread, there have been reports of extrapulmonary manifestations of the disease. Cardiovascular manifestations of COVID-19 are diverse and include acute coronary syndrome, myocarditis masquerading as ST-segment elevation myocardial infarction, acute pericarditis with pericardial effusion, heart failure, arrhythmias, and thromboembolic events [2,3]. This case report describes a patient who had minimal symptoms for weeks and was found to have large pericardial effusion.

## Case presentation

**Figure 1:**
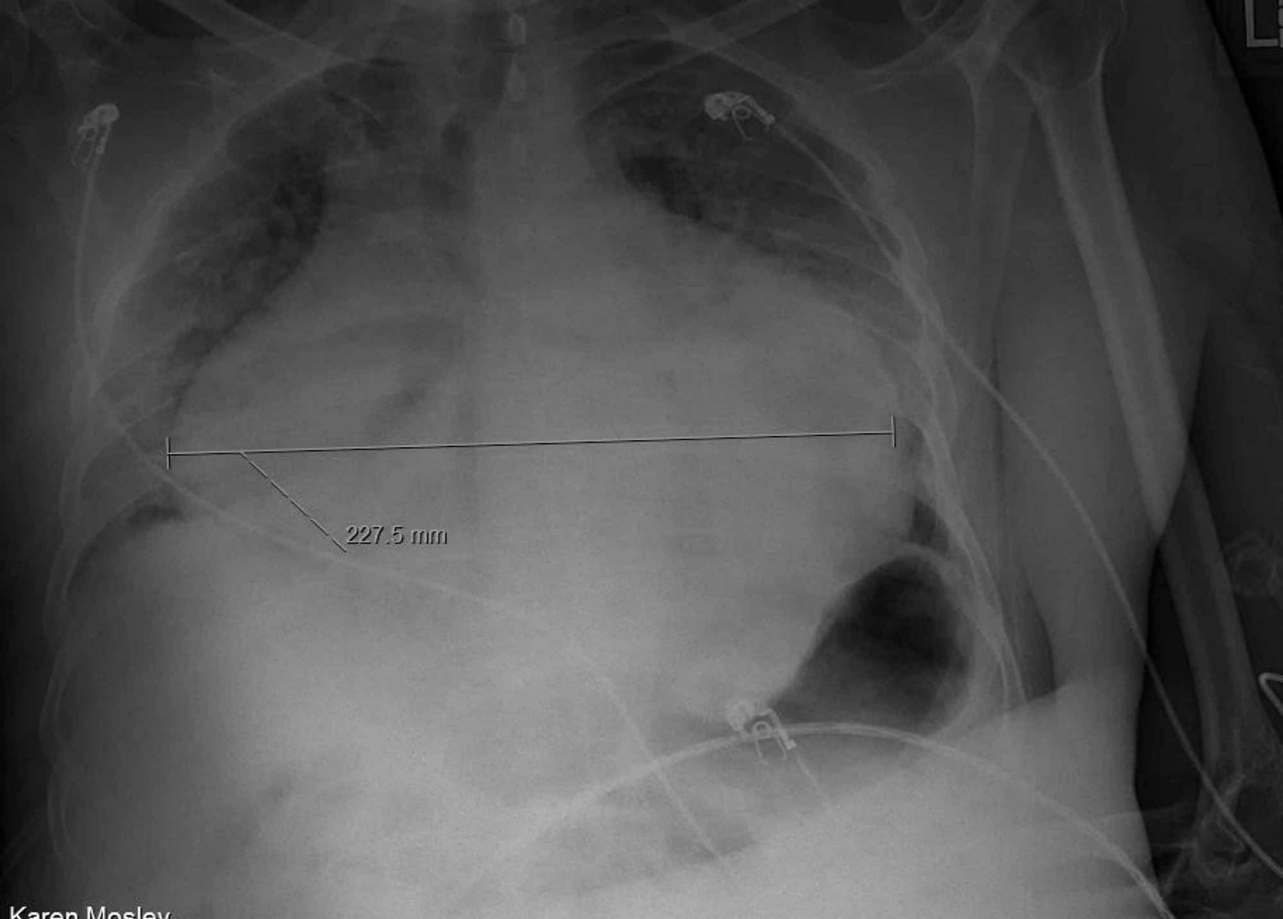
Chest X-ray shows cardiomegaly with diffuse bilateral infiltrates

A 54-year-old male patient with no past medical history presented with complaints of mild intermittent chest pain for weeks. Vital signs on admission blood pressure (BP) 111/72 mmHg, heart rate (HR) 92 beats/minute, respiratory rate (RR) 20 breaths/minute, Temp 97.7°F, and O_2_ saturation of 50%. ECG showed non-specific ST abnormalities. Troponin was negative. Severe acute respiratory syndrome coronavirus 2 (SARS-CoV-2) RNA qualitative nucleic acid amplification test (NAAT) result was positive. The initial chest x-ray revealed cardiomegaly with diffuse bilateral infiltrates (Figure 1).

**Figure 2:**
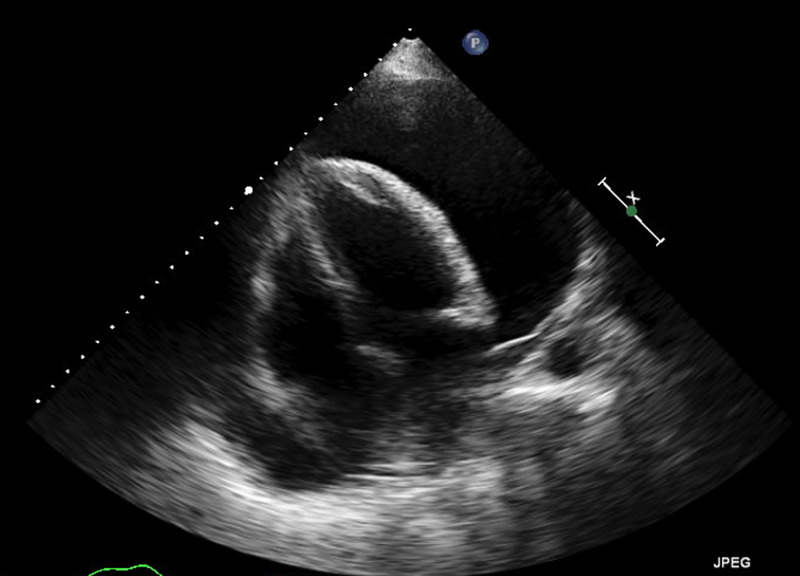
Transthoracic echocardiogram shows large pericardial effusion.

A bedside echocardiogram was performed as a result of chest X-ray findings to evaluate for pericardial effusion. 2-D echocardiogram revealed marked decreased left ventricular ejection fraction (LVEF) with large pericardial effusion (Figure 2). 

Interventional radiology (IR)-guided pericardiocentesis drained a total of 1,500 cc of transudative pericardial fluid. The initial fluid was serosanguineous and, subsequently, serous. Fluid cytology showed 46% lymphocytes and 49% macrophages. Fluid, viral, fungal, and acid-fast bacteria (AFB) culture results were negative. The patient was managed on non-invasive positive pressure ventilation (NPPV), steroids, and hydroxychloroquine for the management of COVID-19. Post-pericardiocentesis echocardiogram showed an improved LVEF of 50%-55%. Although having a long hospitalization course, the patient improved and was discharged for outpatient follow-up. 

## Discussion

SARS-CoV-2 is a novel virus that causes COVID-19 disease. SARS-CoV-2, like other viral infections, can cause pericarditis and resulting pericardial effusion; however, it is usually entailing an acute course. It is postulated that SARS-CoV-2 can cause an exaggerated inflammatory response that may lead to pericardial inflammation and pericardial effusion [4-6]. The pericardial sac typically holds up to 50 mL of fluid of plasma ultrafiltrate for lubrication. Pericardial effusion is generally defined as an increase in pericardial fluid volume. Pericardial fluid can increase to 200 mL in acute disease and up to 2,000 mL in subacute and chronic conditions without resulting in tamponade physiology.

This can manifest as mild subacute or chronic symptoms. The severity of the presenting symptoms influences the patient's drive to seek medical assistance. This, in turn, results in late diagnosis and prolonged hospital course. Diagnosis is established by clinical suspicion at first, which is then confirmed by echocardiography, which helps in the detection of the effusion, estimate the size, and assess the hemodynamic status of the effusion. Elevated cardiac enzymes and wall motion abnormalities on echocardiogram point towards myopericarditis and myocardial involvement. ECG findings in patients with pericardial effusion are sinus tachycardia, low QRS voltage, and electrical alternans. Elevated jugular venous pressure and pulsus paradoxus are suggestive of cardiac tamponade. Echocardiography plays an important role in the diagnosis of cardiac tamponade. The echocardiographic features of cardiac tamponade include collapse of the right atrium at end diastole and the right ventricle in early diastole, reciprocal changes in left and right ventricular volumes with respiration, which are important in the pathogenesis of pulsus paradoxus, increased respiratory variation of mitral and tricuspid valve inflow, dilation (plethora) of the inferior vena cava, and less than a 50% reduction in its diameter during inspiration [7,8]. Patients with large effusions or effusions associated with hemodynamic instability and tamponade require urgent drainage. Fluid analysis typically reveals exudative effusion. RT-PCR COVID-19 of pericardial fluid has been used as a diagnostic aid. Treatment of pericarditis with small effusion using colchicine and corticosteroids has been suggested, but no current guidelines are available [9]. A high index of suspicion and early identification of pericardial effusion allows prompt management of this rare extrapulmonary complication of COVID-19.

## Conclusions

COVID-19 is associated with many cardiovascular complications including pericarditis and acute or subacute pericardial effusion with or without tamponade physiology. Subacute effusion can have an indolent course. Hence, a high degree of suspicion and early intervention are necessary to prevent further complications like cardiac tamponade, which will lead to hemodynamic collapse and cardiac arrest if not identified early. More research is needed including imaging studying and postmortem histological analysis to understand the mechanism of cardiac complications of COVID-19 as well as the association with pericardial effusion, confirm the diagnosis, and determine treatment options.

## References

[1] Guan WJ, Ni ZY, Hu Y (2020). Clinical characteristics of coronavirus disease 2019 in China. N Engl J Med.

[2] Khalid N, Chen Y, Case BC (2020). COVID-19 (SARS-Cov-2) and the heart: an ominous association. [Epub ahead of print]. Cardiovasc Revasc Med.

[3] Su YB, Kuo MJ, Lin TY, Chien CS, Yang YP, Chou SJ, Leu HB (2020). Cardiovascular manifestation and treatment in COVID-19. J Chin Med Assoc.

[4] Inciardi R, Lupi L, Zaccone G (2020). Cardiac involvement in a patient with coronavirus disease 2019 (COVID-19). JAMA Cardiol.

[5] Dabbagh MF, Aurora L, D'Souza P, Weinmann AJ, Bhargava P, Basir MB (2020). Cardiac tamponade secondary to COVID-19. JACC Case Rep.

[6] Aghagoli G, Gallo Marin B, Soliman LB, Sellke FW (2020). Cardiac involvement in COVID-19 patients: risk factors, predictors, and complications. A review. J Card Surg.

[7] Vakamudi S, Ho N, Cremer PC (2017). Pericardial effusions: causes, diagnosis, and management. Prog Cardiovasc Dis.

[8] Purohit R, Kanwal A, Pandit A (2020). Acute myopericarditis with pericardial effusion and cardiac tamponade in a patient with COVID-19. Am J Case Rep.

[9] Sauer F, Dagrenat C, Couppie P, Jochum G, Leddet P (2020). Pericardial effusion in patients with COVID- 19: case series. [Epub ahead of print]. Eur Heart J Case Rep.

